# Self-Healing Polymeric Puerarin Hydrogel Dressing Promotes Diabetic Wound Healing Through Synergistic Immunomodulation and Tissue-Regenerative Remodeling

**DOI:** 10.3390/bioengineering12040427

**Published:** 2025-04-18

**Authors:** Shaohui Geng, Li Liu, Mureziya Yimingjiang, Zhimin Lin, Jingyuan Fu, Shasha Yu, Xinxin Li, Aimin Yan, Kai Yuan, Guangrui Huang, Anlong Xu

**Affiliations:** 1 School of Life Science, Beijing University of Chinese Medicine, Beijing 100029, China20210221036@bucm.edu.cn (S.Y.); 20220931153@bucm.edu.cn (A.Y.); yuankai@bucm.edu.cn (K.Y.); 2 School of Acupuncture and Moxibustion and Massage, Beijing University of Chinese Medicine, Beijing 100029, China; 19801210724@163.com; 3 School of Chinese Pharmacy, Beijing University of Chinese Medicine, Beijing 100029, China; 20220221007@bucm.edu.cn; 4 National Key Laboratory of Efficacy and Mechanism on Chinese Medicine for Metabolic Diseases, Beijing Research Institute of Chinese Medicine, Beijing University of Chinese Medicine, Beijing 102488, China; lixinxin@bucm.edu.cn

**Keywords:** tissue engineering, puerarin, hydrogel, wound healing, polymeric biomaterials, biocompatibility, tissue regeneration, wound dressings

## Abstract

Chronic wound healing is a significant challenge in diabetes. Puerarin is an active compound extracted from the traditional Chinese medicine *Pueraria lobata*. Puerarin has been used in the treatment of diabetes and derives benefits from its antioxidant, anti-inflammatory, antibacterial, and pro-angiogenesis properties, but its efficacy is hampered by poor water solubility and bioavailability. In this study, we designed a polyvinyl alcohol (PVA)–borax–puerarin (BP) hydrogel system that self-assembled via boronic ester bonds. The BP hydrogel exhibited exceptional physical characteristics, including adaptability, injectability, plasticity, self-healing capabilities, and robust compressive strength, as well as good biocompatibility. In the chronic wound diabetic rats model, the BP hydrogel significantly accelerated wound healing, as evidenced by hematoxylin and eosin (HE) staining, as well as Masson and picrosirius red (PSR) staining. RNA–sequencing and multiple immunohistochemistry (mIHC) analyses revealed that the BP hydrogel exerts a therapeutic effect by modulating macrophage polarization, promoting angiogenesis, and regulating collagen remodeling. Our findings suggest that the BP hydrogel represents a promising wound dressing and holds great potential for clinical applications in acute and chronic wound management.

## 1. Introduction

It is estimated that the global diabetes patient population has soared to 828 million, with 80% of these cases concentrated in low- and middle-income countries [1,2,3,4]. Diabetic chronic wounds represent the most prevalent complication of diabetes, affecting between 60% and 80% of patients. The healing of skin wounds progresses through four distinct stages: hemostasis, inflammation, proliferation, and remodeling [5]. Based on the duration of the healing cycle, wounds are categorized as either acute or chronic, with diabetic wounds being a prototypical example of chronic wounds [6,7]. Chronic wounds trigger local chronic micro-inflammation, peripheral vascular disease, neuropathy, persistent inflammation, wound infection, and other conditions [8], ultimately leading to reduced granulation tissue formation, impaired angiogenesis at the wound site, and a sluggish or even stagnant healing process. Thus, diabetic wound healing is a formidable challenge, and there is an urgent need for effective treatment strategies [9].

Traditional wound dressings, such as gauze, films, and bandages, have limited functions. Their primary roles are physical barriers and absorbents for wound exudates, but they are inadequate in addressing the intricate pathological mechanisms underlying delayed healing in diabetic wounds [10]. The use of traditional natural medicines for external application in diabetes wound healing boasts a long history. However, a major challenge faced by numerous flavonoids present in traditional Chinese medicine is their low solubility [11,12], which undermines the drug’s efficacy. Puerarin (P), an 8-C-glucoside compound derived from the traditional Chinese herb *Pueraria lobata*, exhibits antioxidant, anti-inflammatory, antibacterial, and angiogenic properties [13,14]. Puerarin is used in the treatment of diabetes and its complications, which can enhance insulin receptor sensitivity, and reduce protein glycosylation. However, the poor water solubility of puerarin hampers its effectiveness [15,16,17]. The hydrogel preparations have been introduced as a novel approach to enhancing the bioavailability of puerarin [18,19,20]. Hydrogels could increase the solubility of insoluble drugs, improve drug delivery efficiency, enable controlled drug release, and boost overall bioavailability.

Meanwhile, puerarin possesses exceptional gelling capabilities [21]. Because of the unique rigid skeletal structure and multiple modifiable sites, puerarin can self-assemble into a gel without modification or create an interpenetrating network with other substances to enhance gel properties, making it suitable for diabetes wound treatment. Medicinal borax (B), a crystal refined from natural borax minerals, is used externally to clear heat, detoxify, reduce swelling, and prevent corrosion [22,23]. Borax exhibits astringent and protective effects by contracting tissues to reduce secretions through its mild astringent action, forming a physical protective barrier against infection while utilizing its weakly alkaline cleansing properties to remove necrotic tissue and stimulating epithelial cell regeneration to accelerate wound healing [24,25,26]. Additionally, borax frequently serves as a cross-linking agent for gel formation, improving polymer performance by forming dynamic borate ester bonds with polymers, thereby granting materials self-healing properties [27,28,29]. The PVA is a material noted for its excellent biocompatibility and gelling properties [30,31,32]. Given that borax can react with flavonoids possessing an o-dihydroxy or similar structure, in this study we build upon the classic polyvinyl alcohol–borax hydrogel system, and find that the PVA–borax–puerarin hydrogel has the best performance. Furthermore, we formulated the hydrogel into a clinically applicable form, providing a preliminary foundation for advancing puerarin–based therapies in wound management.

## 2. Materials and Methods

### 2.1. Preparation of Puerarin–Borax–PVA (BP) Hydrogel

The BP hydrogel was prepared through a two–step grinding process. Initially, a 10 wt% PVA solution was created by dissolving 10 g of PVA powder (P914585, Shanghai Macklin, Shanghai, China) in deionized water heated to 95 °C. Next, puerarin (S30646, Shanghai Yuanye, Shanghai, China; at concentrations of 1, 2, or 4 wt%) was dispersed in a 2 wt% borax (S24099, Shanghai Yuanye, Shanghai, China) solution in deionized water at 25 °C using ultrasound (Kunshan Shumei KQ3200DE, Wuhu, China). Subsequently, 10 mL of this puerarin–borax mixture was added to 10 mL of the PVA solution and mixed with a glass rod to form a hydrogel. The hydrogel was then allowed to swell for 24 h at room temperature.

### 2.2. Molecular Structure and Physical Characterization of Hydrogel

#### 2.2.1. Fourier Transform Infrared Spectroscopy (FTIR)

The freeze-dried substances were weighed in appropriate quantities, and their infrared spectra were analyzed using an FTIR spectrometer (Bruker VERTEX 70, Bruker Optics GmbH & Co. KG, Ettlingen, Germany). The measurement parameters employed in this study included a spectral range from 4000 to 400 cm^−1^, a spectral resolution of 4 cm^−1^, and 32 cumulative scans per spectrum, with air used as the background for spectral comparison.

#### 2.2.2. Scanning Electron Microscope (SEM)

The surface of the hydrogel samples and the brittle fracture surfaces obtained after treatment with liquid nitrogen were scanned using a field emission scanning electron microscope (ZEISS-SUPRA55, Carl Zeiss GmbH, Oberkochen, Germany). Additionally, the samples were suspended, droplet-casted onto tin foil, dried under a UV lamp, and gold-coated for further observation. Finally, the images were viewed under an accelerating voltage of 5 kV.

#### 2.2.3. Rheological Properties

The rheological characteristics of the hydrogels were evaluated using an Anton Paar MCR 302 rheometer. Dynamic strain scans were conducted to assess the variations in the storage modulus (G′) and loss modulus (G″) of the hydrogel within both the critical strain region and the linear viscoelastic range. Specifically, at a frequency of ω = 10 rad/s, the dynamic strain of the hydrogel was adjusted from 0.01% to 1000%. Furthermore, step-strain scanning was employed to investigate the self-healing capabilities of the hydrogels. This involved alternating the oscillatory strain amplitudes between 1% (maintained for 3 min) and 200% (maintained for 3 min), up to a maximum of 800% strain, to observe the changes in the hydrogel’s properties.

#### 2.2.4. Physical Characterization

To assess the hydrogels’ injectability, moldability, adaptability, self-healing properties, and malleability, several tests were conducted. A syringe was used to evaluate the injectability of the hydrogels, and the injected gel was then shaped into various forms to study its moldability and adaptability. Additionally, the hydrogels were colored with dyes, and segments of different colored hydrogels were cut and spliced together to investigate their self-healing capabilities. To observe the ductility of the hydrogels, they were manually stretched and placed on white paper.

### 2.3. Evaluation of Hydrogel Biocompatibility

#### 2.3.1. Cell Counting Kit-8 (CCK–8) Cell Proliferation Assay

A 96-well plate was loaded with 100 μL of L929 cell suspension (1.5 × 10^4^ cells per well) and pre-incubated for 24 h at 37 °C with 5% CO_2_. Under aseptic conditions, the hydrogel was prepared and introduced into the plate along with 0.01 g/mL of extraction solution. The plate was then incubated for 72 h, with the experiment divided into a control group, a hydrogel group, and 5 replicate wells for each group. Subsequently, 10 μL of CCK-8 solution was added to each well, and the plate was further incubated at 37 °C for 1–4 h. Afterward, 100 μL of the reaction solution was transferred to a new 96–well plate, and the absorbance at 450 nm was measured using an enzyme counter. The control group consisted of hydrogels without inoculated cells.

Cell survival rate = [(A_s_ − A_b_)/(A_c_ − A_b_)] × 100%

A_s_: Absorbance of experimental wells (with cells, medium, CCK-8 solution with drugs).

A_c_: Absorbance of control wells (with cells, medium, CCK-8 solution, without drugs).

A_b_: Absorbance of blank wells (with medium, CCK-8 solution, without cells, drugs).

#### 2.3.2. Calcein–AM/PI Live/Dead Cell Staining

The Live/Dead Cell Viability Kit was utilized to stain and assess cell growth on hydrogels after a one-day incubation period, with the complete medium serving as the control. Live cells, stained with calcein–AM, were visualized under a fluorescence microscope using emission filters set at (Ex/Em: 495 nm/520 nm), emitting green fluorescence. Conversely, dead cells, stained with PI, were observed using emission filters set at (Ex/Em: 530 nm/620 nm), emitting red fluorescence.

#### 2.3.3. Hemolysis Test

From each tube, 500 μL of rat blood was collected and centrifuged at 4 °C at a speed of 3500 rpm for 5 min. The erythrocytes settled at the bottom were resuspended in 5 mL of saline and then centrifuged three more times. Subsequently, 250 mg of hydrogel samples were placed in tubes containing 1 mL of erythrocytes and allowed to incubate at room temperature for 1 h. To determine the degree of hemolysis, comparisons were made with a negative control (saline) and a positive control (Triton X–100). After centrifuging all samples at 3500 rpm for 5 min, the supernatant was collected, and its absorbance at 545 nm was measured using an enzyme marker. The hemolysis rate for each hydrogel proportion was calculated, and samples with a hemolysis rate below 5% were deemed to comply with international standards, indicating favorable blood biocompatibility.

Hemolysis rate (%) = (uptake_sample_ − negative control uptake)/(positive control uptake − negative control uptake) × 100%

#### 2.3.4. Antibacterial Experiment of Hydrogel

A total of 10 mL of *Staphylococcus aureus* [10^6^ colony forming units CFU)/mL] was added to the sterilized broth medium. The hydrogels (1 mL) were taken and inoculated into the medium, and an equal amount of the medium was added to the control group. After shaking at 37 °C for 24 h, the mixture was diluted by 10^6^ and coated on a solid broth medium. After incubation at 37 °C for 20 h, the number of colonies on the agar plate was counted. The results were expressed as the percentage of killing, and the test was repeated three times.

### 2.4. Evaluation of the Efficacy of Hydrogel for Wound Healing

#### 2.4.1. The Chronic Wound Model in Diabetic Rats

Experiments were conducted using 72 male Sprague-Dawley rats (4–6 weeks old, 250 ± 10 g) bred under SPF conditions. These rats were randomly (using the random number method) divided into four groups: the model group, the P group (treated with puerarin and petroleum jelly), the B group (treated with B hydrogel), and the BP group (treated with BP hydrogel), with 18 rats in each group. The experiment was approved by the Ethics Committee of the Beijing University of Chinese Medicine under the approval number BUCM-2022060103-2135 and was conducted at the animal experiment center of the university.

To establish the chronic wound model, we prepared a streptozotocin (STZ) solution and injected it intraperitoneally into the fasted rats within 30 min of preparation, ensuring that the STZ was kept in an ice bath and wrapped in aluminum or tin foil to maintain its stability. The rats were fasted for 14 h prior to the injection. Fasting blood glucose levels were measured daily via tail vein blood collection starting 72 h after the STZ injection. The modeling was considered successful when the blood glucose level stabilized at 16.7 mmol/L. After successfully establishing the diabetic model, we created full-thickness incisions on both the left and right sides of the rats’ spines, positioned 2 cm away from the spine and extending to the subcutaneous layer. The diameter of each incision was approximately 8 mm.

#### 2.4.2. Treatment Protocol

The model group received standard wound care, which included daily iodophor disinfection, wound protection with petroleum jelly, and dressing with a combination of petroleum jelly gauze and plain gauze. Additionally, the P group received an ointment mixture containing 1% puerarin and petroleum jelly, the B group received B hydrogel (B–PVA, without puerarin), and the BP group received BP hydrogel (B–PVA, with puerarin). The dosage for each group was adjusted to ensure complete wound coverage. The healing progress of the wounds was documented on days 3, 7, and 10 to compare the therapeutic efficacy among the groups.

#### 2.4.3. Evaluation of In Vivo Wound Healing

On days 3, 7, and 10 following the establishment of the wound model, photographs of the wound were captured. The actual trauma area was then measured using Image–J software (version 1.53a, *n* = 12, six rats per group). The wound healing rate was determined by the formula [(original area − measured area)/original area] × 100%, where the original area refers to the actual trauma area on the day the skin trauma model was created.

#### 2.4.4. Histological Analysis

Following treatment, the skin tissues of rats were preserved using polyformaldehyde, subsequently dehydrated, embedded in paraffin, and sectioned longitudinally along the skin near the wounds. These sections were then stained using HE and Masson’s trichrome staining to analyze the pathological structure and collagen fiber distribution within the healing tissues.

### 2.5. The Mechanism of BP Hydrogel in Promoting Wound Healing

#### 2.5.1. RNA-Sequencing

On the 10th day of treatment in the chronic wound models, three rats were randomly chosen from each group. Skin samples were collected from the wound areas, immediately placed in liquid nitrogen for rapid freezing, and then transferred to a −80 °C refrigerator for storage. These samples were later sent to Shanghai Meiji Biomedical Technology Co. for RNA-seq gene sequencing analysis.

RNA was extracted from the tissue samples, and its concentration and purity were assessed using a Nanodrop2000 instrument. The integrity of the RNA was verified through agarose gel electrophoresis, while the RNA integrity number (RIN) values were determined using an Agilent 2100 system. Sequencing libraries for each RNA sample were prepared by following the manufacturer’s instructions using the Ion total RNA-Seq Kit v2 (Life Technologies, Carlsbad, CA, USA). Subsequently, the cDNA libraries were sequenced on an Illumina NovaSeq 6000 platform according to standard sequencing protocols.

The transcriptome sequencing data were statistically analyzed utilizing the Meiji Cloud Platform (https://cloud.majorbio.com/). The volcano diagram of differential gene expression (DEGs) analysis was conducted using the DESeq2 software package (Version 1.24.0) [33]. Goatools (Version 1.4.4, https://github.com/tanghaibao/GOatools) was employed for GO enrichment analysis on the genes within the gene set. For KEGG pathway enrichment analysis, custom R scripts were used to analyze the genes in the gene set, following the same principle as GO function enrichment analysis. The statistical significance was set at *p*-value < 0.05, and the single differential gene expression analysis was considered a *p*-adjust (FDR-adjusted *p*-values using the Benjamini–Hochberg method) < 0.05 as statistical significance.

#### 2.5.2. Multiple Immunohistochemistry (mIHC)

For multiple immunohistochemistry, we employed the following primary antibodies: Anti-VEGFA (abcam, ab1316, diluted 1:100), Anti-CD31 (Abcam, ab182981, diluted 1:500), Anti-Mannose Receptor (CD206) (Abcam, ab64693, diluted 1:500), Anti-iNOS (Abcam, ab210823, diluted 1:500), Anti-Collagen I (Abcam, ab270993, diluted 1:500), and Anti-Collagen III (Abcam, ab184993, diluted 1:100).

Slices were selected from various stages, including the inflammatory, proliferating, and remodeling phases. Each tissue section was then incubated with an appropriate amount of primary antibody of suitable concentration (diluted in antibody diluent) to ensure complete tissue coverage. The sections were placed in a humidified chamber and incubated overnight at 4 °C (alternatively, for 2 h at room temperature or 30 min at 37 °C). Following this, the secondary antibody was diluted to the appropriate concentration and added dropwise to the slide, ensuring adequate coverage of the tissues. Subsequently, DAPI staining solution was added dropwise to the tissues and stained for 5 min at room temperature. Finally, the slides were sealed with an anti-fluorescence quencher.

### 2.6. Statistical Analysis

The statistical analysis of all data in this study was conducted using SPSS 22.0 and GraphPad Prism 10 software. Measurement data were presented as the mean ± standard deviation (x ± s), while count data were presented as the number of cases (*n*). For data conforming to a normal distribution, multifactor ANOVA and the chi-square test were employed. In cases of non-conformity to normal distribution, a nonparametric test was used. When comparing multi-sample means, repeated-measures ANOVA was applied. For count data, the χ^2^ test was utilized. A *p*-value of less than 0.05 was considered statistically significant. *p*-adjust (FDR-adjusted *p*-values using the Benjamini–Hochberg method) was used to analyze the single difference of genes on the Majorbio Cloud Platform.

## 3. Results and Discussion

### 3.1. Preparation and Characterization of the BP Hydrogel

**Figure 1:**
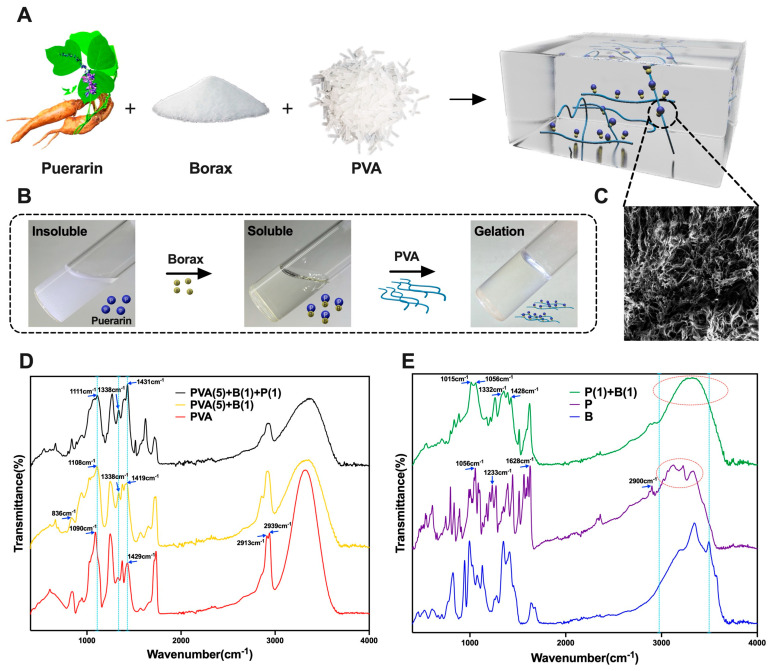
Schematic diagram of raw materials, production process, and reaction principle required for BP hydrogel. Note: (**A**) Raw materials for preparing puerarin–borax–PVA hydrogel; (**B**) The apparent characteristics of puerarin–borax solution and BP hydrogel; (**C**) SEM structure of PB–PVA hydrogel; (**D**) FTIR spectra of PVA, borax–PVA, borax–puerarin–PVA; (**E**) FTIR spectra of puerarin, borax, and puerarin–borax.

The wound dressing was established by a two–step manufacturing process aiming to produce a borax–puerarin–PVA hydrogel (Figure 1A). Puerarin and borax were mixed to enhance puerarin’s solubility via self-assembly at room temperature (Figure 1B), and we observed the pore size using scanning electron microscopy (SEM) (Figure 1C).

Borax was added into the puerarin solution to boost puerarin’s solubility in water. Approximately one molecular weight of borax dissolved two molecular weights of puerarin (Table S1, Figure S1). We hypothesized that borax could cross-link with hydroxyl groups to form borate ester bonds. Then, puerarin’s water solubility could be improved.

The Fourier Transform Infrared (FTIR) spectrum of PVA in Figure 1D revealed specific absorption peaks. It included the O–H stretching vibration (*ν*_OH_) at 3320 cm^−1^, asymmetric stretching vibrations (*νas*_CH2_) at 2939 cm^−1^, symmetric stretching vibrations (*νs*_CH2_) at 2913 cm^−1^, bending vibration (*δ*_CH2_) at 1429 cm^−1^, and C–O bond stretching vibration (*ν*_C-O_) at 1090 cm^−1^. In contrast, the borax hydrogel exhibited characteristic peaks indicative of borate species, such as the asymmetric stretching vibration of the B–O–C bond at 1419 cm^−1^, 1338 cm^−1^, and 1108 cm^−1^, respectively [34,35]. In the BP hydrogel, the asymmetric stretching vibrations of B–O–C bonds were observed at 1431 cm^−1^, 1338 cm^−1^, and 1111 cm^−1^, respectively. This phenomenon may be attributed to the enhanced ionic bonding interactions within the B–O–C linkages induced by the incorporation of puerarin, resulting in a slight shift of the characteristic peaks toward higher wavenumbers. Notably, based on cross-linking PVA with borax, the O–H stretching vibration peak of PVA transforms formed a sharp to a broader and shorter shape, confirming successful hydrogel formation.

Also, the results showed (Figure 1E) that in puerarin, stretching vibrations of O–H at 3200–3400 cm^−1^ (*v*_OH_), stretching vibrations of saturated alkyl groups at 2900 cm^−1^ (*v*_CH_), stretching vibrations of C=O bonds near 1628 cm^−1^ (*v*_C=O_), and stretching vibrations of C-O bonds on phenolic hydroxyl and sugar hydroxyl groups near 1233 cm^−1^ and 1056 cm^−1^ (*v*_C-O_) can be observed. After mixing puerarin with borax, the sharp O–H stretching vibration absorption peaks of phenols and alcohols at 3340 cm^−1^ and 3495 cm^−1^ of puerarin changed to a wide peak at 3311 cm^−1^, and the C–O bond stretching vibration at 1056 cm^−1^ also shifted towards low wavenumbers, forming a double peak at 1015 cm^−1^ and 1056 cm^−1^. The absorption peak at 1428 cm^−1^ and the shoulder peak at 1332 cm^−1^ indicate the formation of B–O–C bonds. This indicates that a coordination bond has been formed between the hydroxyl group on the sugar group of puerarin and the borate ion through cross-linking.

### 3.2. Rheological Behavior and Physical Properties of BP Hydrogel

The borax–PVA hydrogel is a well-established biocompatible hydrogel [28,36,37]. Borax acts as an effective cross-linking agent to facilitate the gelation of PVA in aqueous solutions, producing a hydrogel with high water content. This hydrogel exhibited excellent biocompatibility, indicating a promising candidate for use as a wound dressing in skin-healing applications. However, the physical properties were characterized by high brittleness, limited elasticity, inability to stretch, poor healing capacity, and weak resistance to external forces. After a series of experiments, we found that adding puerarin to the system of borax–PVA hydrogel can significantly improve its physical properties in a certain proportion. Furthermore, the optimal gel composition was determined to be 1 wt% borax, 1 wt% puerarin, and 5 wt% PVA (Figure S2). The specific ratio and physical characteristics of this optimized hydrogel are shown in Table S2.

**Figure 2:**
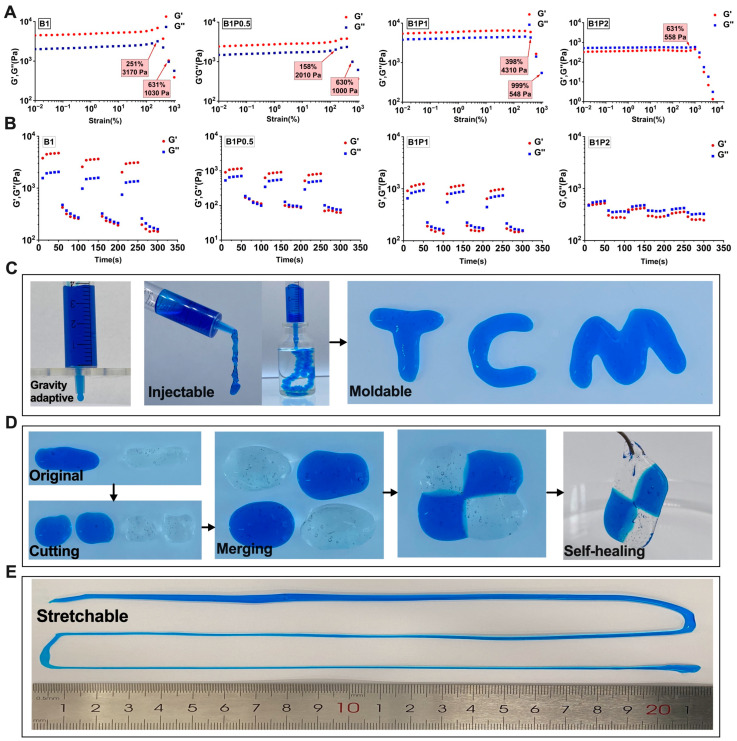
Evaluation of physical properties of BP hydrogel. Note: (**A**) G′ and G′′ of PB hydrogel at different concentrations under strain amplitude; (**B**) G′ and G′′ of BP hydrogel under alternate scanning; (**C**) Gravity adaptation of hydrogel (it can flow out of the syringe under the action of gravity), injectability and plasticity (various letters and patterns can be made from the injected hydrogel); (**D**) Self-adaptation and self-healing properties of hydrogels (the hydrogel pieces can approach each other through adaptability and re-fuse into a new hydrogel); (**E**) Ductility (the hydrogel can stretch the strip to adapt to the external pressure to better protect the wound).

Then, the rheological test was conducted to demonstrate the properties of the BP hydrogel (Figure 2A). The B_1_, B_1_P_0.5_, and B_1_P_1_ hydrogels exhibited gel-like behavior (G′ > G″) within the linear viscoelastic region. As the amplitude increased, G′ and G″ intersected, indicating a transition to a fluid state when G′ < G″. Conversely, the B_1_P_2_ hydrogel remained in a fluid state (G′ < G″) throughout the linear viscoelastic region. There was no intersection point observed between G′ and G” as the amplitude increased. The B_1_, B_1_P_0.5_, and B_1_P_1_ hydrogels displayed shear-thinning characteristics, albeit with varying shear storage moduli. With increasing puerarin concentration, the extent of the linear viscoelastic zone initially decreased. Later, the extent of the linear viscoelastic zone increased. The linear viscoelastic zone gradually expanded by increasing puerarin concentration, accompanied by changes in G′max. Among these formulations, B_1_P_1_ exhibited the longest linear viscoelastic zone and the highest G′max with superior adaptability (398%) and strength (4310 Pa). These properties enabled B_1_P_1_ to resist external damage while maintaining colloidal characteristics. In contrast, B_1_P_2_ possessed a wide linear viscoelastic range (631%) with strength significantly decreased to 558 Pa. This could be attributed to the formation of borate bonds and numerous hydrogen bonds between puerarin and borax enhancing the adaptability and strength of the hydrogel up to a certain concentration. The alternating shear-thinning test revealed the hydrogel exhibited colloidal properties at low strain (1%) (G′ > G′′) and fluid properties at high strain (800%) (G′ < G′′) (Figure 2B). The hydrogel’s initial state could be restored after transitioning from high to low strain, indicating excellent self-healing properties.

Then, the optimized BP hydrogel (5 wt% PVA, 1 wt% borax, 1 wt% puerarin) was usd to test its physical properties. First, the BP hydrogel demonstrated remarkable adaptability. It could be filled into a syringe cavity by gravity and naturally drip out (Figure 2C). Injectability was confirmed by the hydrogel’s capacity to be extruded from the syringe needle under pressure. Then, a continuous strip could be formed. Moldability was defined by the hydrogel’s ability to be molded into specific shapes, maintaining this shape for an extended period. These properties rendered the hydrogel a viable material for accommodating wound dressings of various shapes. As depicted in Figure 2D, the hydrogel possessed exceptional self-healing characteristics. The hydrogel promptly fused back into a single, intact entity after dividing into two segments and re-contacting. This phenomenon is attributed to the numerous borate bonds within the BP hydrogel, which undergo continuous dissociation and re-polymerization. It could impart self-healing properties to the hydrogel. These self-healing capabilities ensured the durability of the gel for external applications. Furthermore, the hydrogel exhibited impressive ductility, capable of being stretched to nearly one meter in length (initial volume is about 1 cm^3^), as shown in Figure 2E. It could provide favorable conditions for wound dressings.

### 3.3. Cell and Blood Biocompatibility of BP Hydrogel

**Figure 3:**
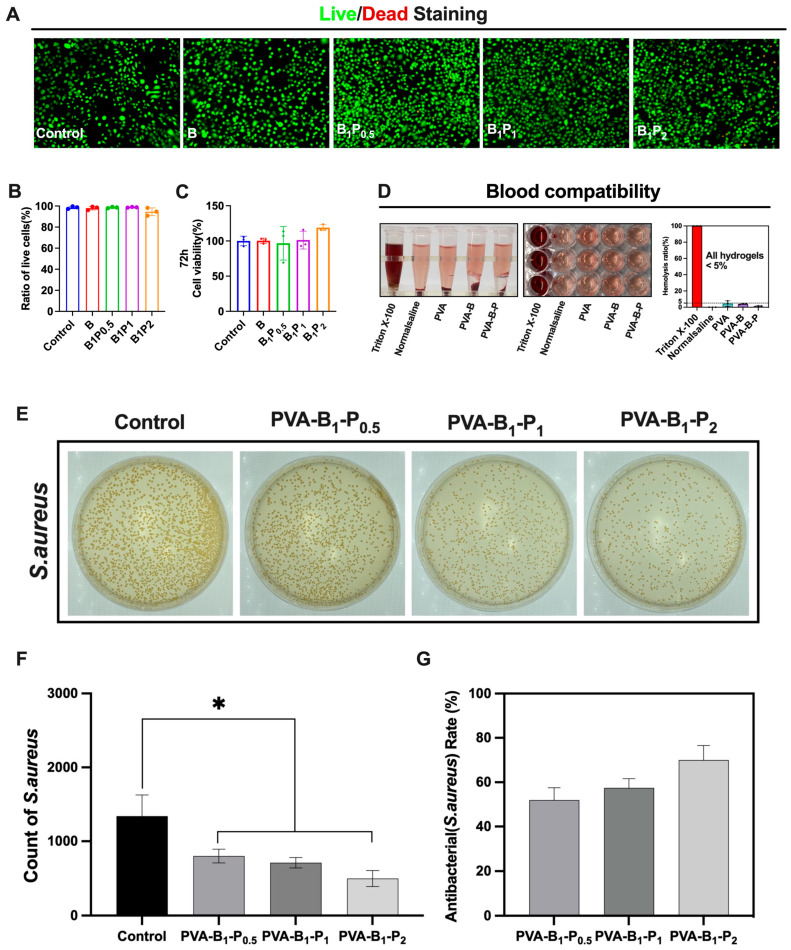
Biocompatibility of BP hydrogel. Note: (**A**,**B**) Live and dead staining of L929 when co-culture with BP hydrogel; (**C**) CCK–8 test of BP hydrogel (*n* = 3); (**D**) Detection of BP hydrogel hemolysis test and comparison of hemolysis rate; (**E**) Photo of BP hydrogel inhibiting S.a.; (**F**) The comparison of S.a. count of BP hydrogel (*n* = 3); (**G**) The antibacterial rate of BP hydrogel (*n* = 3). * *p* < 0.05.

The mouse fibroblast cell line (L929) served as a standard model employed in biocompatibility assessments [38,39,40]. Given the direct contact between the hydrogel and skin tissue, the cytotoxicity of the hydrogel could be assessed through co-culture with skin fibroblasts. In this study, we utilized cell staining and CCK–8 detection methods to evaluate the cytotoxicity of the hydrogel. During the cell staining, the entire field of vision for the co-culture of hydrogel and cells at various concentrations was dominated by green, indicating viable cells. This observation suggested that the co-culture of hydrogel and fibroblasts exhibited good cytocompatibility (Figure 3A,B). Furthermore, the CCK–8 experiment after 72 h of co-culture revealed that the survival rate of BP hydrogel at low and medium concentrations was nearly 100%. With an increase in puerarin concentration, a proliferative effect on cell growth was observed (Figure 3C). Collectively, these findings indicated that BP hydrogel could possess a favorable biocompatibility and demonstrated the ability to promote cell proliferation.

A crucial aspect of wound dressings was used to evaluate blood compatibility. Typically, the hemolysis rate would not exceed 5% under the international ASTM F756-17 standard [41,42,43]. In this study, we assessed the hemolytic properties of PVA, B hydrogel, and BP hydrogel with rat blood as the evaluation sample. The hemolysis rate of BP hydrogel in this study was found to be less than 5% (Figure 3D). This result indicated that BP hydrogel exhibited good blood compatibility and national standards for wound dressings.

Then, the antibacterial effects of BP hydrogel was also evaluated. As shown in Figure 3E,F, a large number of strains can be seen on the bacterial coating plate of the control group, and the number of bacteria on the plate of the gel group is significantly less than that of the control group. The bacteriostatic effect of the gel formulation demonstrated a concentration-dependent correlation with elevated puerarin levels (Figure 3G), indicating that the BP hydrogel dressing exhibited significant inhibitory effects on Staphylococcus aureus growth. This antimicrobial property establishes a mechanistic foundation for its therapeutic potential in diabetic wound healing.

### 3.4. BP Hydrogel Promoted the Healing of Chronic Wounds in Diabetic Rats

**Figure 4:**
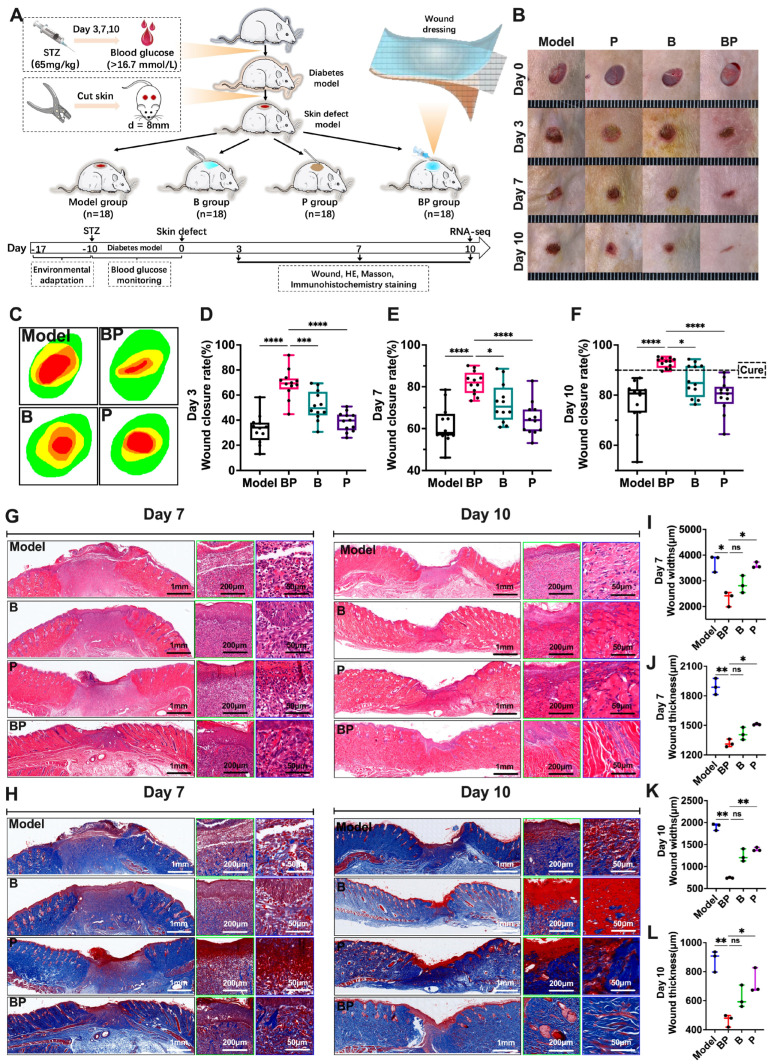
The effect of BP hydrogel for promoting the healing of chronic wound healing in diabetes. Note: (**A**) Design of the experiment; (**B**,**C**) Comparison pictures of wound healing at different time points; (**D**) The comparison of wound healing rate at Day 3 (*n* = 12); (**E**) The comparison of wound healing rate at Day 7 (*n* = 12); (**F**) The comparison of wound healing rate at Day 10 (*n* = 12); (**G**) The HE staining of wound tissue at Day 7 and 10; (**H**) The Masson staining of wound tissue at Day 7 and 10; (**I**–**L**) The comparison of wound widths and thickness at Day 7 and 10 (*n* = 3). * *p* < 0.05, ** *p* < 0.01, *** *p* < 0.001, **** *p* < 0.0001, ns: no significance.

We employed hydrogel for the treatment of diabetic wounds. The experimental design for the application of hydrogel in the treatment of chronic diabetic wounds is illustrated in Figure 4A.

As depicted in Figure 4B–D, the wound healing rates observed in the B hydrogel and BP hydrogel groups surpassed the model group on the third day. Specifically, the B hygrogel and BP hydrogel groups exhibited a statistical significance in promoting wound healing (*p* < 0.01) and P group (*p* < 0.01). Conversely, the P group demonstrated no significant effect on wound healing. The wound healing rates of the B hydrogel and BP hydrogel groups remained higher than those of the model group on the seventh day (Figure 4F). Both groups showed a statistically significant effect on promoting wound healing (*p* < 0.01) and the P group (*p* < 0.01). The P group did not have an effect on wound healing promotion.

Pathological observations of skin healing tissue confirmed that BP hydrogel had the effect of accelerating wound healing. At the inflammatory stage (third day, Figure S3A,B), the model group rats exhibited skin epithelial defect with minimal and uneven collagen production. In contrast, the BP hydrogel group showed a significantly higher degree of epithelization (Figure S3C,D). During the proliferation stage (seventh day, Figure 4G,I,J), all treatment groups exhibited accelerated epithelialization, with the BP hydrogel group demonstrating superior wound closure efficacy. Notably, this cohort showed a concurrent reduction in defect width and granulation tissue thickness, accompanied by prominent neovascularization observable through high-power microscopic examination. Collagen was neatly arranged (Figure 4H). At the remodeling stage (tenth day, Figure 4G,H), the BP hydrogel group formed complete epithelial tissue with numerous new blood vessels and hair follicles. Collagen was evenly distributed. The model group’s skin healing tissue had large defects, incomplete epithelization, and uneven collagen distribution. The healing process of the model group’s skin was significantly slower than BP hydrogel group (Figure 4K,L).

Thus, BP hydrogel significantly enhanced the wound-healing–promoting effect of puerarin and outperformed B hydrogel wound dressing. Therefore, it represented an ideal material to facilitate chronic wound healing in diabetes.

### 3.5. RNA–Seq Revealed the Mechanism of Hydrogel Promoting Wound Healing

**Figure 5:**
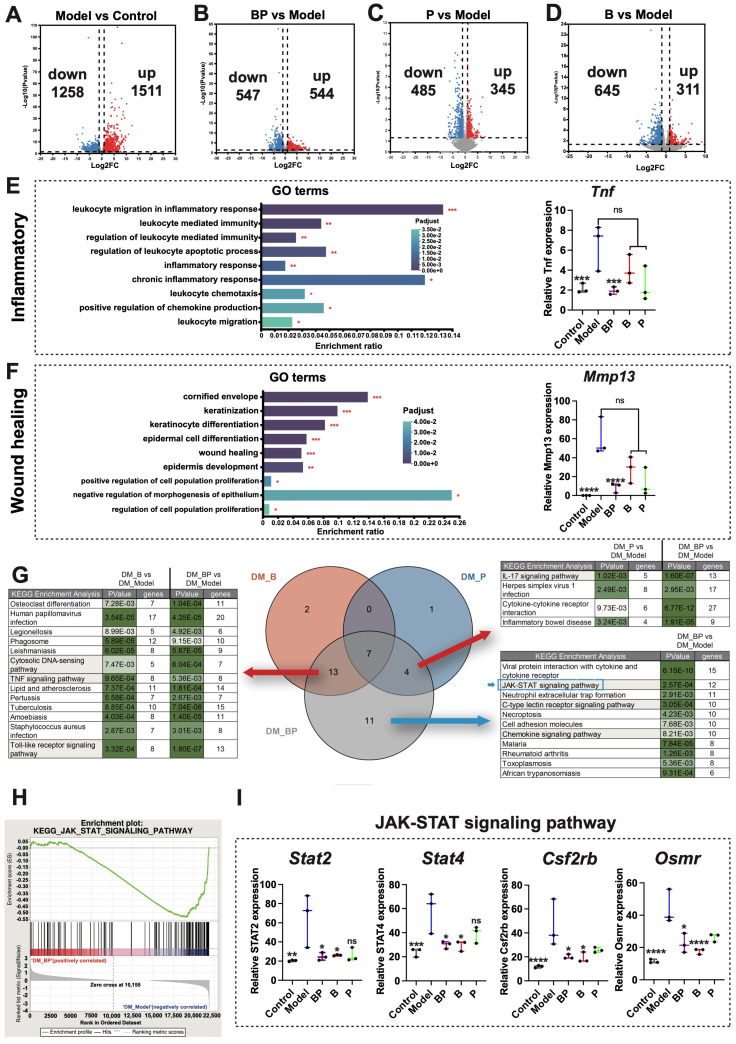
RNA–Seq analysis of BP hydrogel in promoting wound healing. Note: (**A**) The volcano diagram of DEGs between model and control group; (**B**) The volcano diagram of DEGs between BP and model group; (**C**) The volcano diagram of DEGs between P and model group; (**D**) The volcano diagram of DEGs between B and model group; (**E**) The GO enrichment terms about inflammatory of DEGs between BP and model group; (**F**) The GO enrichment terms about wound healing of DEGs between BP and model group; (**G**) The KEGG enrichment of DEGs between four groups; (**H**) The GSEA analysis of JAK–STAT signaling pathway between BP and model group; (**I**) The DEGs of JAK–STAT signaling pathway between four groups (*n* = 3). * *p-adjust* < 0.05, ** *p-adjust* < 0.01, *** *p-adjust* < 0.001, **** *p-adjust* < 0.0001, ns: no significance.

To investigate the mechanism of BP hydrogel in accelerating wound healing, we used RNA–Seq to analyze the gene expression profiles at skin defect sites. Compared with the normal group, the model group exhibited a total of 2769 differentially expressed genes (DEGs), comprising 1511 upregulated and 1258 downregulated genes (Figure 5A and Figure S4A). In contrast, the BP hydrogel group, relative to the model group, demonstrated 1091 DEGs, with 544 upregulated and 547 downregulated genes (Figure 5B). Additionally, the P group identified 830 DEGs (345 upregulated and 485 downregulated; Figure 5C), while the B hydrogel group exhibited 956 DEGs (311 upregulated and 645 downregulated; Figure 5D). The heatmap was constructed based on the DEGs between four groups (Figure S4B). The control group displayed significant differences compared with the model group. Moreover, the differential gene expression pattern in the BP hydrogel group and the control group exhibited similarity to group P and group B, suggesting that the BP hydrogel group exhibited a higher degree of skin healing.

To elucidate the biological functions of these DEGs, Gene Ontology (GO) analysis was performed using gene annotation and functional databases (Figure 5E). DEGs were categorized into three stages including inflammation, proliferation, and remodeling. Specifically, the inflammatory stage included inflammation-related pathways such as “inflammatory response” and “chronic inflammatory response”. This also included immune cell-related terms such as “leukocyte migration in inflammatory response”, “leukocyte mediated immunity”, “regulation of leukocyte mediated immunity”, and “leukocyte migration”. Among them, *Tnf* genes were important cytokines to promote the inflammatory differentiation of macrophages [13,44]. The model group was significantly higher than the control group (*p* < 0.001), and the BP hydrogel group reduced its expression (*p* < 0.001). P group did not show statistical differences, reflecting the unique effect of BP hydrogel.

The pathways associated with wound healing included the stages of proliferation and collagen remodeling. As shown in Figure 5F, the proliferation stage was represented by “regulation of cell population proliferation”, “keratinocyte differentiation”, “epidermal cell differentiation”, and “positive regulation of cell population proliferation”. Furthermore, the remodeling phase encompassed terms like “epidermis development”, “keratinization”, and “cornified envelope”. MMP–13 was associated with wound healing, which was produced by fibroblasts in chronic skin ulcers, and its expression increased during the excessive inflammation phase [45]. However, it was not expressed in normally healing adult skin wounds [46]. The high expression state in the model group indicated the delayed healing state of the model group. The model group was significantly higher than the normal group (*p* < 0.0001). The BP hydrogel group reduced its expression (*p* < 0.0001), while the P group did not show statistical differences. These findings indicated that BP hydrogel could facilitate the entire wound healing process.

To identify the key signaling pathways associated with these DEGs, pathway analysis was conducted with the Kyoto Encyclopedia of Genes and Genomes (KEGG) database. The Venn diagram of KEGG pathways among Groups B, P, and BP was generated (Figure 5G). Notably, two KEGG pathways were exclusive to Group B, while one pathway was unique to Group P. A total of 11 KEGG pathways were exclusive to the BP group, including the JAK–STAT signaling pathway, which was closely associated with wound healing. Continuous JAK–STAT activation could promote chronic inflammation [47,48]. The JAK–STAT signaling pathway was activated in the model group, with the *Stat2*, *Stat4*, *Csf2rb*, *Osmr*, *IL19*, *Irf9*, *IL24*, and *IL21r* expression significantly higher than normal (Figure 5I and Figure S5). In contrast, the expression level in the BP hydrogel group was close to that of the normal group, demonstrating a highly healed state. Additionally, Gene Set Enrichment Analysis (GSEA) was conducted to identify key signaling pathways, revealing the “JAK–STAT signaling pathway”, “toll–like receptor signaling related to Myd88”, and “cytokines and inflammatory response” (Figure 5H and Figure S4C,D). These findings suggested that BP hydrogel could promote wound healing by modulating these pathways.

### 3.6. BP Hydrogel Enhanced Anti-Inflammatory, Angiogenesis, and Collagen Remolding in the Process of Wound Healing

To gain insights into the mechanisms of BP hydrogel to promote wound healing, our investigation centered on three pivotal processes. The process included the regulation of macrophage polarization in the inflammatory phase, the augmentation of angiogenesis in the proliferative phase, and the modulation of collagen remodeling in the remodeling phase [49].

**Figure 6:**
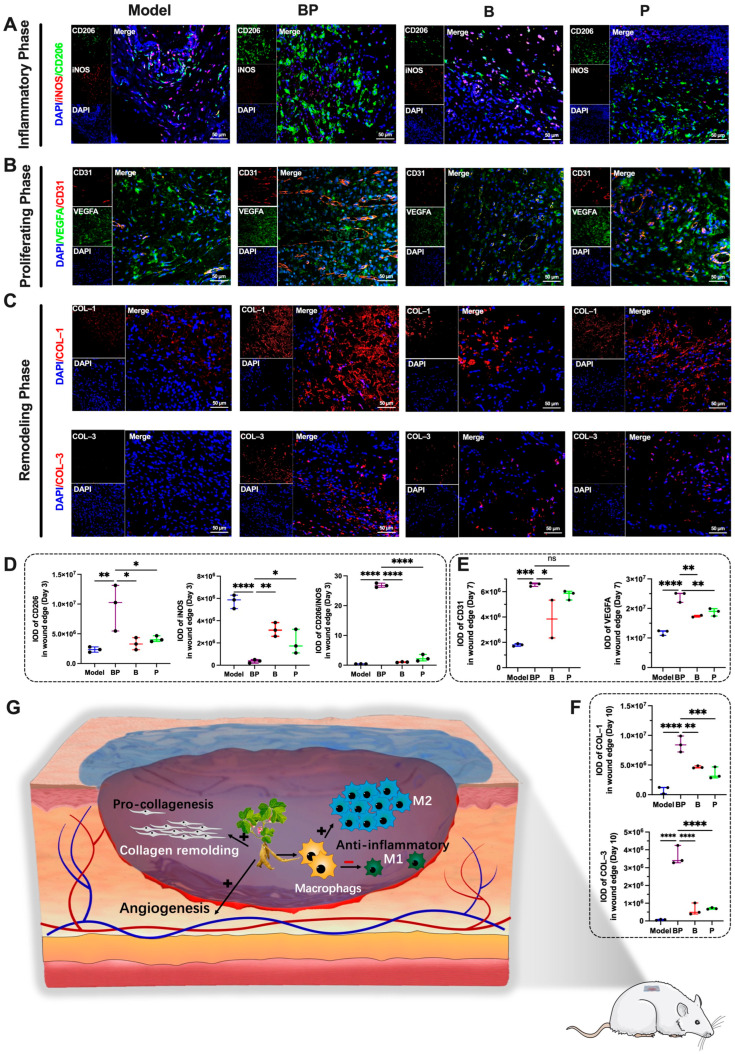
Investigation of the mechanisms underlying the wound healing promotion by BP hydrogel based on mIHC. Note: (**A**) The expression of CD206 and iNOS in wound area (*n* = 3); (**B**) The expression of VEGFA and CD31 in wound area (*n* = 3); (**C**) The expression of COL–1 and COL–3 in wound area (*n* = 3); (**D**) The IOD of CD206 and iNOS in wound area (*n* = 3); (**E**) The IOD of VEGFA and CD31 in wound area (*n* = 2–3); (**F**) The IOD of COL–1 and COL–3 in wound area (*n* = 3); (**G**) The mechanism diagram of BP hydrogel promoting wound healing. * *p* < 0.05, ** *p* < 0.01, *** *p* < 0.001, **** *p* < 0.0001, ns: no significance.

The initial stage of wound healing was marked by skin inflammation [50,51]. Diabetic wounds exhibited a prolonged inflammatory phase to delay the healing process. Macrophages serve as the primary inflammatory cells during the inflammatory phase of skin healing [52]. These cells differentiated into pro-inflammatory (M1, Type 1 macrophages) and anti-inflammatory (M2, Type 2 macrophages) phenotypes based on the microenvironment. M1 macrophages secreted inflammatory cytokines exhibiting the capacity to eliminate pathogenic microorganisms. Conversely, M2 macrophages secrete cytokines to foster angiogenesis and cellular proliferation [53,54]. As illustrated in Figure 6A,D, on the third day, BP hydrogel significantly elevated the polarization expression of M2 macrophages (*p* < 0.01) and decreased the expression of M1 macrophages (*p* < 0.0001). This outcome underscored BP hydrogel’s efficacy in mitigating early wound inflammation. These results depicted the mechanism to diminish the inflammatory state during the wound’s inflammatory phase.

Revascularization of wounds was a pivotal aspect of the healing process and facilitated accelerated recovery. Vascular endothelial growth factor (VEGF) stimulated endothelial cell proliferation and migration. It could accelerate angiogenesis and provide essential nutrients [55,56,57]. Additionally, CD31 played a significant role in the physiological processes of wound healing and angiogenesis [58,59,60]. As illustrated in Figure 6B,E, on the 7th day post-treatment, the results demonstrated that BP hydrogel significantly enhanced the expression of CD31 and VEGFA at the wound site. Specifically, the expression levels of CD31 in the BP hydrogel group were significantly higher than those in the model group (*p* < 0.001) and the B group (*p* < 0.05). Similarly, the expression of VEGFA in the BP group was significantly elevated compared with the model group (*p* < 0.0001) and the B group (*p* < 0.01). Furthermore, the VEGFA expression in the BP group was significantly higher than that in the P group (*p* < 0.01). These findings indicated that BP hydrogel could promote wound healing by stimulating angiogenesis.

Type I collagen was the predominant collagen type, providing robust structural support to skin wounds [61,62]. Type III collagen plays a pivotal role in skin wound healing, closely associated with the elasticity and scarring of newly formed skin [63,64]. As illustrated in Figure 6C,F, immunohistochemical analysis revealed that BP hydrogel significantly promoted the remodeling of both collagen types during the remodeling phase. Specifically, the model group exhibited low Type I collagen content at the wound site, whereas the B group and P group increased Type I collagen expression (*p* < 0.01, *p* < 0.001, respectively). The BP hydrogel group demonstrated substantial Type I collagen production (*p* < 0.001). Quantitative analysis indicated that the Type I collagen content in the BP hydrogel group was significantly higher than that in the B group and P group (*p* < 0.05). A similar trend was also observed in Type III collagen, with the BP group exhibiting higher levels than the B group and P group (*p* < 0.05). These findings suggested that BP hydrogel could promote wound healing by facilitating collagen repair at the wound site.

Overall, BP hydrogel exhibited significant efficacy to enhance wound healing by the inflammatory phase, the proliferative phase, and the collagen remodeling phase. These findings underscored the multifaceted mechanisms by which BP hydrogel facilitates wound healing. It could be a promising therapeutic option for wound management.

## 4. Conclusions

The objective of this study was to develop a natural hydrogel dressing suitable for clinical use. From a screen of Chinese herbal medicine monomers with antioxidant properties, puerarin was selected and formulated into a hydrogel alongside borax and PVA. The formulation not only improved puerarin’s water solubility via borax-mediated solubilization but also optimized the hydrogel’s physical characteristics by competitively binding with PVA, transforming brittle matrices into adaptive materials with self-healing properties ideal for clinical wound dressings, while simultaneously enhancing therapeutic efficacy in chronic wound healing through bioactive compound delivery.

Physical testing revealed that the BP hydrogel possessed exceptional physical characteristics, including a wide linear viscoelastic range, robust compressive strength, and self-healing capabilities. It also demonstrated remarkable adaptability, injectability, moldability, and ductility. The biocompatibility assessments showed that BP hydrogel exhibits excellent biocompatibility with fibroblast cell line and blood cells. Moreover, the BP hydrogel demonstrated superior wound healing efficacy in diabetic rats. These results indicate a promising potential of the BP hydrogel as dressing in the treatment of external wounds.

We also investigated the therapeutic mechanisms of the BP hydrogel on wound healing using RNA–Seq and mIHC. The efficacy of BP hydrogel was evident throughout the entire wound healing process, as it modulated macrophage polarization during the inflammatory phase, promoted angiogenesis during the proliferative phase, and regulated collagen remodeling during the remodeling phase.

In conclusion, while BP hydrogel demonstrates significant advantages as an easily prepared wound dressing with exceptional physical properties and therapeutic efficacy, several challenges require resolution prior to clinical translation. The long-term biosafety profile of borax-containing formulations warrants further toxicological evaluation, and industrial-scale production feasibility needs verification through pilot manufacturing trials. Future research should explore synergistic combinations with other bioactive phytochemicals to enhance multifunctionality. From a commercialization perspective, the cost-effective raw materials and simple preparation protocol position this hydrogel as a promising candidate for industrial development, pending rigorous assessment of shelf-life stability and sterilization compatibility under manufacturing conditions.

## Data Availability

The data that support the findings of this study are available from the corresponding author upon reasonable request.

## Supplementary Materials

The following supporting information can be downloaded at: https://www.mdpi.com/article/10.3390/bioengineering12040427/s1, Table S1: Research on the ratio of borax to improve the water solubility of puerarin; Table S2: Description of the state and physical characteristics of hydrogels with different ratios; Figure S1: Photos of borax improving the water solubility of puerarin; Figure S2: RNA–Seq analysis of BP hydrogel in promoting wound healing; Figure S3: The DEGs involved in JAK–STAT pathway; Figure S4: RNA–Seq analysis of BP hydrogel in promoting wound healing. A. The Venn diagram of four groups. B. The heatmap diagram of DEGs among four groups. C. The GSEA analysis of Toll–like receptor signaling related to Myd88 between BP and model group. D. The GSEA analysis of Cytokines and inflammatory response between BP and model group; Figure S5: The DEGs involved in JAK–STAT pathway. A. The IL29 expression among four groups (*n* = 3). B. The Irf9 expression among four groups (*n* = 3). C. The IL24 expression among four groups (*n* = 3). D. The IL21r expression among four groups (*n* = 3). * *p* < 0.05, ** *p* < 0.01, *** *p* < 0.001, **** *p* < 0.0001.

## Abbreviations

The following abbreviations are used in this manuscript:

HE	hematoxylin and eosin
PSR	picrosirius red
mIHC	multiple immunohistochemistry
P	puerarin
B	borax
BP	puerarin–borax–PVA
PVA	polyvinyl alcohol
SEM	scanning electron microscopy
FTIR	Fourier Transform Infrared
L929	the mouse fibroblast cell line
DEGs	differentially expressed genes
GO	Gene Ontology
KEGG	Kyoto Encyclopedia of Genes and Genomes
GSEA	Gene Set Enrichment Analysis
M1	Type 1 macrophages
M2	Type 2 macrophages
CCK-8	Cell counting Kit-8
STZ	streptozotocin
RIN	RNA integrity number
CD206	Anti-Mannose Receptor
