# Supplementary material for: Self-Healing Polymeric Puerarin Hydrogel Dressing Promotes Diabetic Wound Healing Through Synergistic Immunomodulation and Tissue-Regenerative Remodeling
Source: Bioengineering (Basel). 2025 Apr 18;12(4):427. doi: 10.3390/bioengineering12040427 (PMC12024557; doi:10.3390/bioengineering12040427)
Supplement: Supplementary file 1 [file bioengineering-12-00427-s001.zip › bioengineering-3544722-supplementary.pdf]

Supporting Information

Self-Healing Polymeric Puerarin Hydrogel Dressing Promotes Diabetic Wound Healing Through Synergistic Immunomodulation and Tissue-Regenerative Remodeling

Shaohui Geng [1], Li Liu [1], Mureziya Yimingjiang [1], Zhimin Lin [2], Jingyuan Fu [3], Shasha Yu [1,3], Xinxin Li [4], Aimin Yan [1], Kai Yuan [1], Guangrui Huang [1,*] and Anlong Xu [1,*]

[1] School of Life Science, Beijing University of Chinese Medicine, Beijing 100029, China;
[2] School of Acupuncture and Moxibustion and Massage, Beijing University of Chinese Medicine,
Beijing 100029, China;
[3] School of Chinese Pharmacy, Beijing University of Chinese Medicine, Beijing 100029, China;
[4] National Key Laboratory of Efficacy and Mechanism on Chinese Medicine for Metabolic Diseases,
Beijing Research Institute of Chinese Medicine, Beijing University of Chinese Medicine,
Beijing 102488, China;
* Correspondence: hgr@bucm.edu.cn (G.H.); xuanlong@bucm.edu.cn (A.X.)

1.1 The apparent characteristics of puerarin borax solution

By adding mineral traditional Chinese medicine borax (sodium tetraborate decahydrate) to the aqueous solution of puerarin, it was found that borax can significantly improve the solubility of puerarin in water. The experimental data are shown in **Table S1** and **Figure S1**. Among them, a 1 wt% aqueous solution of puerarin has obvious needle like precipitates. After adding 0.5 wt% or 1 wt% borax and thoroughly mixing, the puerarin solution becomes clear. The aqueous solution of 2 wt% puerarin has obvious needle like precipitates. After adding 0.5 wt% borax and thoroughly mixing, a portion of puerarin dissolves while there is still some precipitation; After adding 1 wt% borax and thoroughly mixing, all puerarin is dissolved and the solution becomes clear.

Table S1. Research on the ratio of borax to improve the water solubility of puerarin

Soluble / Borax	Puerarin		
	0.5 wt%	1 wt%	2 wt%
0 wt%	×	×	×
0.5 wt%	√	√	×
1 wt%	√	√	√

Note: ×: Not completely dissolved, with obvious precipitation; √: Completely dissolved, solution clear and transparent.

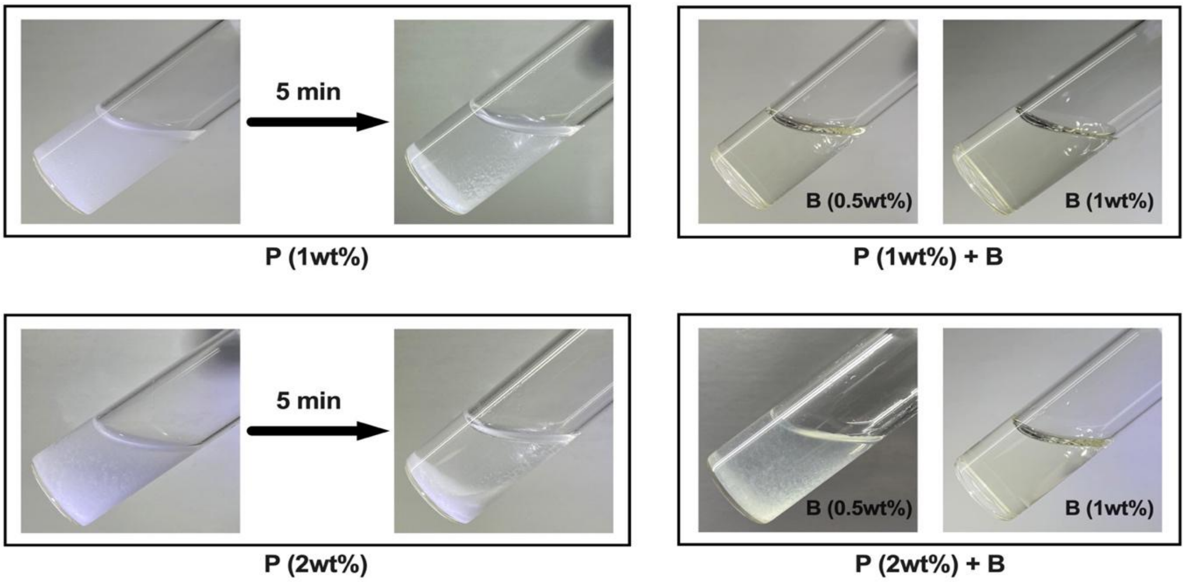

Figure S1. Photos of borax improving the water solubility of puerarin.

Note: Puerarin is insoluble in water and forms a flocculent precipitate after standing still. After adding different concentrations of borax, puerarin gradually dissolves and the solution becomes clear and transparent.

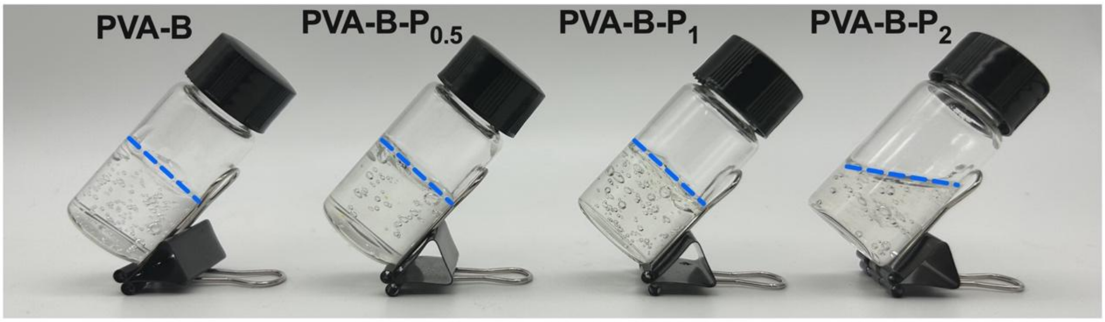

Figure S2. Photos of the prepared hydrogel.

1.2 Physical characterization of PB-PVA hydrogel

As showed in **Table S2**, By adding puerarin to B-PVA hydrogel system, with the increase of puerarin concentration, the physical properties gradually improved. But as the concentration exceeds 2wt%, the system gradually changes from gel state to fluid state.

Table S2. Description of the state and physical characteristics of hydrogels with different ratios

	PVA	Borax	Puerarin	State	Characterization
B_1	5 wt%	1 wt%	0 wt%	Gel	Classic hydrogel, brittle, poor self-healing and ductility
$B_1P_{0.5}$	5 wt%	1 wt%	0.5 wt%	Gel	Brittle texture, improved self-healing and extensibility, but not ideal
B_1P_1	5 wt%	1 wt%	1 wt%	Gel	Ideal self-healing, extensibility, and adhesiveness
B_1P_2	5 wt%	1 wt%	2 wt%	Fluid	Excessive fluidity, not suitable for use as skin dressings

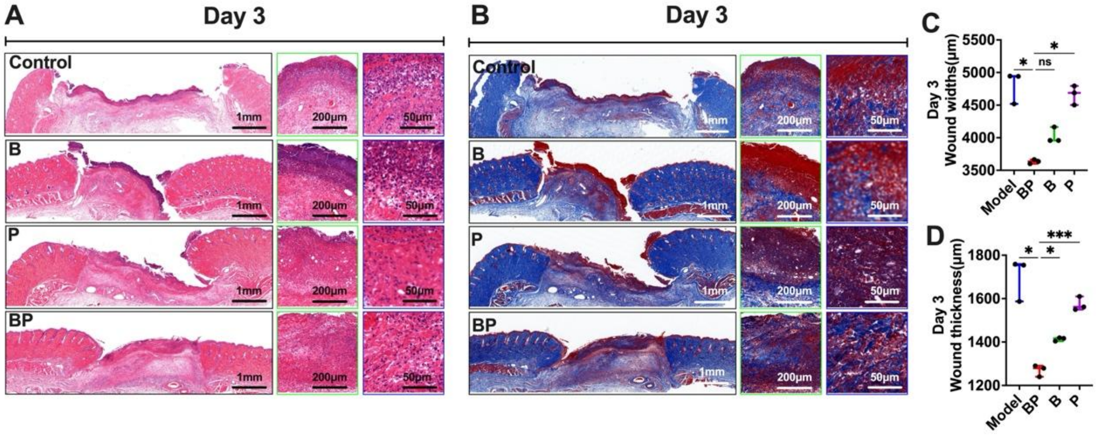

Figure S3. The effect of BP hydrogel for promoting the healing of chronic wound healing at Day 3. Note: A. The HE staining of wound tissue at Day 3; B. The Masson staining of wound tissue at Day 3; C,D. The comparison of wound widths and thickness at Day 3 (n=3). *$P < 0.05$, ***$P < 0.001$.

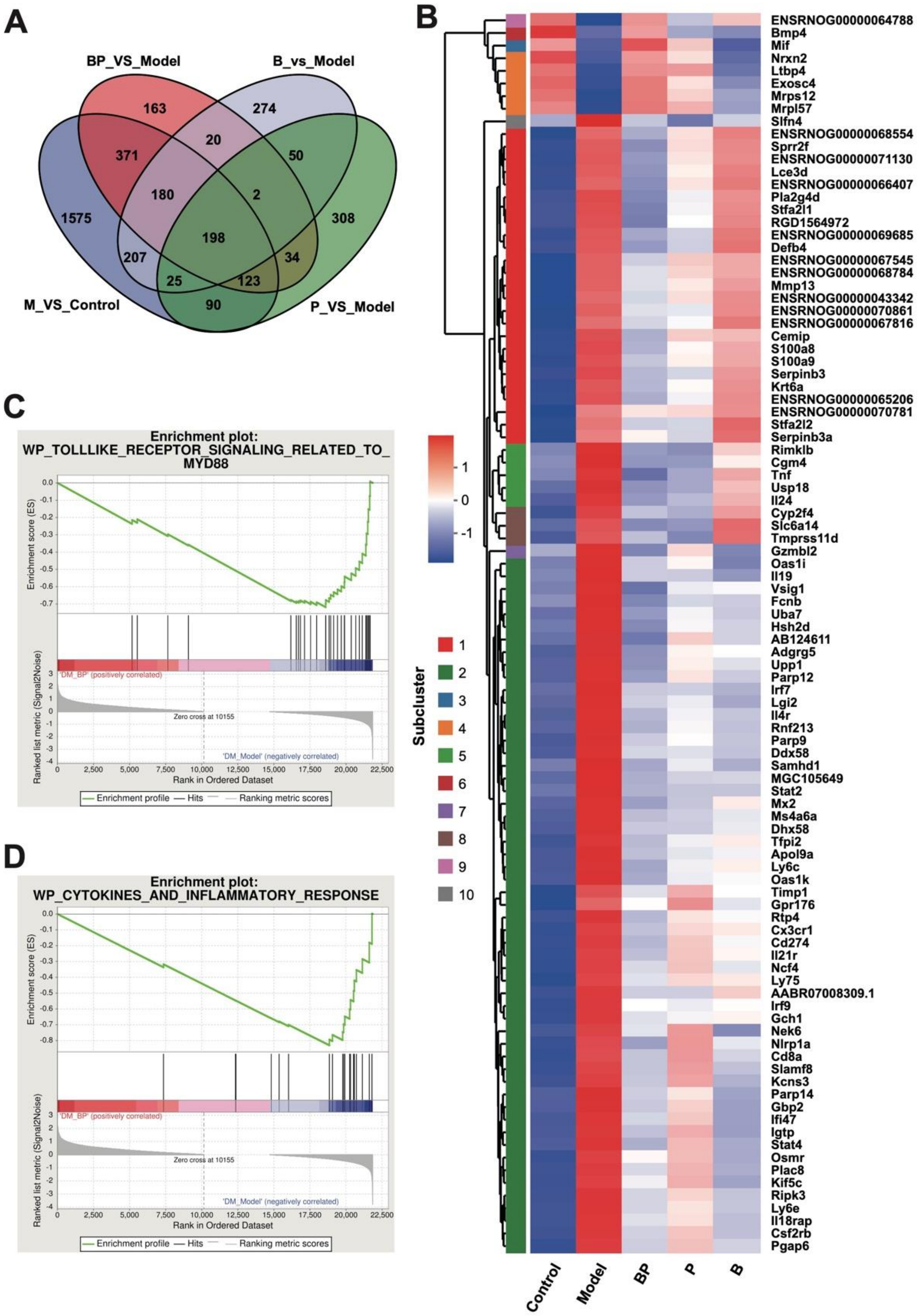

Figure S4. RNA-Seq analysis of BP hydrogel in promoting wound healing.

A. The Venn diagram of four groups. **B.** The heatmap diagram of DEGs among four groups. **C.** The GSEA analysis of Toll-like receptor signaling related to Myd88 between BP and model group. **D.** The GSEA analysis of Cytokines and inflammatory response between BP and model group.

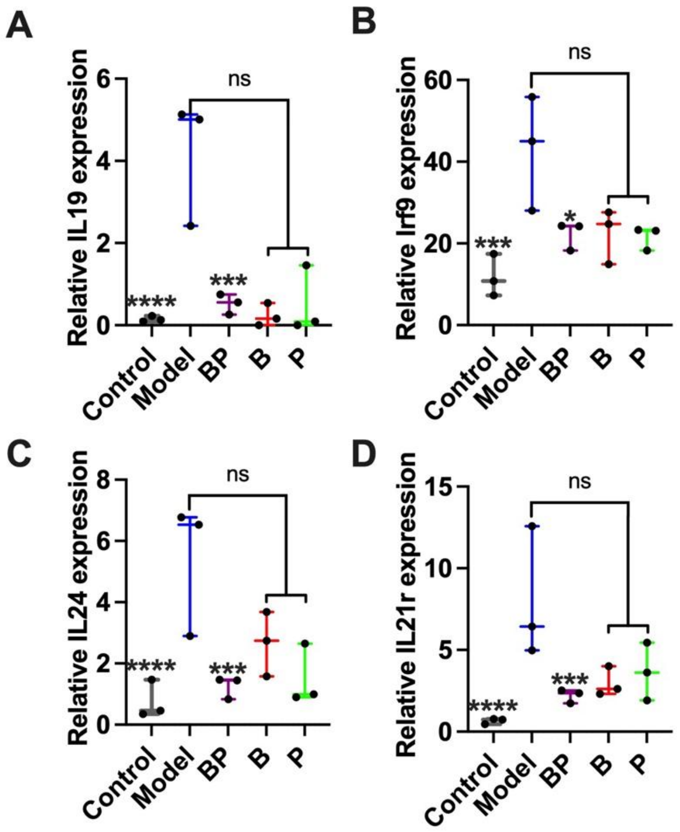

Figure S5. The DEGs involved in JAK-STAT pathway.

A. The IL29 expression among four groups (n=3). **B.** The Irf9 expression among four groups (n=3). **C.** The IL24 expression among four groups (n=3). **D.** The IL21r expression among four groups (n=3). *P < 0.05, **P < 0.01, ***P < 0.001, ****P < 0.0001.
